# Therapeutic vancomycin monitoring: a comparative analysis of high-performance liquid chromatography and chemiluminescent microparticle immunoassay methods in liver transplant recipients

**DOI:** 10.3389/fphar.2025.1516339

**Published:** 2025-06-03

**Authors:** Soha Azadi, Seyed Soroush Jalali, Soliman Mohammadi-Samani, Parisa Ghasemiyeh, Bita Geramizadeh, Hamed Nikoupour, Afsaneh Vazin, Mojtaba Shafiekhani

**Affiliations:** ^1^ Department of Clinical Pharmacy, School of Pharmacy, Shiraz University of Medical Sciences, Shiraz, Iran; ^2^ Shiraz Transplant Center, Abu-Ali Sina Hospital, Shiraz University of Medical Sciences, Shiraz, Iran; ^3^ Department of Pharmaceutics, School of Pharmacy, Shiraz University of Medical Sciences, Shiraz, Iran; ^4^ Pharmaceutical Sciences Research Center, Shiraz University of Medical Sciences, Shiraz, Iran; ^5^ Department of Pathology, Shiraz University of Medical Sciences, Shiraz, Iran; ^6^ Shiraz Transplant Research Center, Shiraz University of Medical Sciences, Shiraz, Iran

**Keywords:** chemiluminescent microparticle immunoassay, high-performance liquid chromatography, therapeutic drug monitoring, vancomycin, infectious disease

## Abstract

**Background:**

Vancomycin is a glycopeptide antibiotic of choice for treating serious Gram-positive bacterial infections, including methicillin-resistant *Staphylococcus aureus* (MRSA). However, its therapeutic efficacy and risk of nephrotoxicity are closely related to maintaining specific serum concentration levels. Liver transplant recipients (LTRs) require precise therapeutic drug monitoring (TDM) due to altered pharmacokinetics. This study compares the accuracy and precision of two vancomycin measurement methods—chemiluminescent microparticle immunoassay (CMIA) and high-performance liquid chromatography (HPLC) in LTRs.

**Methods:**

The cross-sectional study was conducted over 11 months at the Abu-Ali Sina Solid Organ Transplant Hospital in Shiraz, Iran. The study included 34 adult LTRs on vancomycin treatment, excluding those with hypersensitivity, chronic kidney disease, burn injuries, or receiving phenytoin. Blood samples were collected at different intervals post-vancomycin administration and analyzed using both CMIA and HPLC methods.

**Results:**

HPLC demonstrated superior accuracy and precision in measuring vancomycin concentrations, particularly in identifying patients with vancomycin-induced nephrotoxicity. Significantly higher trough (p-value: 0.026) and intermediate (p-value: 0.49) concentrations were detected by HPLC in patients experiencing nephrotoxicity, whereas CMIA did not show significant differences between groups. Pharmacokinetic variables such as half-life (p-value: 0.024) and AUC (p-value:0.037), measured by HPLC, were significantly different between LTRs with and without nephrotoxicity, which was not observed with CMIA.

**Conclusion:**

HPLC is more sensitive and reliable than CMIA for measuring vancomycin levels in LTRs, which is critical for optimizing vancomycin therapy and preventing adverse effects. The research suggests that HPLC should be the preferred method for vancomycin TDM in LTRs and further multicenter studies are recommended to validate these results.

## 1 Introduction

Vancomycin is a glycopeptide antibiotic that is commonly used in the treatment of serious infections caused by Gram-positive bacteria. It is a drug of choice in the management of methicillin-resistant *Staphylococcus aureus* (MRSA) infections, and its therapeutic efficacy is highly dependent on achieving appropriate serum concentrations (Pai et al., 2014). The Chinese Pharmacological Society recommends maintaining a serum trough concentration of vancomycin between 10 and 15 mg/L for adult patients, while a higher range of 10–20 mg/L is advised for serious MRSA infections. Clinical studies indicate that insufficient vancomycin levels can contribute to the emergence of resistance in *S. aureus* strains. Conversely, it is crucial to recognize that achieving a serum trough concentration of 15 mg/L or higher may increase the risk of nephrotoxicity (Ye et al., 2016). Moreover, trough levels alone may not provide a complete picture of the drug’s efficacy or potential for toxicity. Therefore, according to pharmacokinetic (PK)/pharmacodynamic (PD) theory, the better evaluation indicator for therapeutic drug monitoring (TDM) of vancomycin is the ratio of area under the concentration-time curve (AUC) to minimum inhibitory concentration (MIC), which takes into account the entire drug exposure over a dosing interval. To enhance clinical outcomes in the management of invasive MRSA infections, the latest guidelines from the American Society of Health-System Pharmacists (ASHP), Infectious Diseases Society of America (IDSA), and the Society of Infectious Diseases Pharmacists (SIDP) recommended transitioning from trough-based to AUC-guided vancomycin dosing, targeting an AUC/MIC ratio of 400–600 mg h/L based on the MIC of less than 1 mg/L for optimal efficacy against MRSA infections. A loading dose of 20–35 mg/kg is advised, with subsequent dosing adjusted based on renal function (Chen et al., 2022; Rybak et al., 2020; Kaushik et al., 2024). In addition, vancomycin presents a wide interindividual pharmacokinetic variation, a narrow therapeutic index, and the potential for various adverse effects, including nephrotoxicity (Matsumoto et al., 2022). Therefore, TDM of vancomycin and individualized dosing regimens based on patient-specific factors are highly recommended for increasing treatment response while preventing adverse effects (Rybak et al., 2020).

Liver transplant-receiving (LTR) patients are a special population that may require vancomycin therapy due to their immunosuppressed state and increased risk of infections (Morales Junior et al., 2023). However, they also have altered the pharmacokinetics and pharmacodynamics of drugs due to their liver dysfunction, post-operative complications, and polypharmacy (Morales Junior et al., 2023; Righi, 2018). Therefore, accurate TDM of vancomycin is crucial in this population to achieve optimal therapeutic outcomes and avoid toxicity. Studies have shown that TDM of vancomycin in LTRs can improve clinical outcomes, including reducing the incidence of nephrotoxicity and improving bacterial clearance. Additionally, TDM can help prevent the development of antibiotic resistance, being a global challenge (Shafiekhani et al., 2023), by ensuring the appropriate drug use.

Since the primary use of this drug, several methods have been developed and evaluated for the quantification of vancomycin levels in biological fluids, including immunoassays (Yeo et al., 1989; Filburn et al., 1983), high-performance liquid chromatography (HPLC) using either UV detection (Hagihara et al., 2013), fluorescence detection (Abu-Shandi, 2009), or photodiode array detection (Cao et al., 2014), and also liquid chromatography-mass spectrometry (LC-MS) (Oyaert et al., 2015). Up to now, HPLC has been considered the one of gold standards due to its high sensitivity, specificity, and accuracy, wide linear range of detection, low sample requirement, and ability to detect multiple analytes (Cheng et al., 2022; Ningrum et al., 2024). However, it typically involves sample preparation steps, such as protein precipitation or solid-phase extraction, which can be time-consuming and require skilled personnel (Aboelezz et al., 2025).

In recent years, a new method for vancomycin measurement has emerged, known as chemiluminescent microparticle immunoassay (CMIA). This solid-phase immunoassay quantifies vancomycin in serum by detecting the chemiluminescent reaction between the drug and a labeled antibody. CMIA offers several advantages over traditional high-performance liquid chromatography (HPLC), including faster processing times, ease of use, lower costs, and the availability of various commercial kits.

Recent studies have highlighted the effectiveness of CMIA for measuring vancomycin levels, particularly in patients undergoing hemodialysis. For example, a study conducted in Brazil compared CMIA with liquid chromatography coupled with tandem mass spectrometry (LC-MS/MS) and found a strong correlation between the two methods. The mean difference indicated that CMIA provides reliable measurements without cross-reactivity in hemodialysis patients (Scribel et al., 2024). In another advancement, Fan et al. developed an ultra-performance liquid chromatography-tandem mass spectrometry (UPLC-MS/MS) technique to reduce cross-interaction in vancomycin assays. This innovative approach was evaluated against UPLC-UV and CMIA under normal, dialysis, and hemolytic conditions. The results demonstrated a moderate correlation between UPLC-UV and UPLC-MS/MS for samples from dialysis patients; however, some hemolytic samples showed overestimated results when analyzed with CMIA (Fan et al., 2019). The presence of cross-reacting substances, such as the crystalline degradation product of vancomycin, can lead to falsely elevated results in different immunoassay methods. This is particularly concerning in populations with renal impairment, where the accumulation of such metabolites is more likely (Chen et al., 2020).

Comparative studies have assessed CMIA against HPLC for other drugs as well. Guerrero Garduño et al. conducted a comparative analysis of HPLC and CMIA for quantifying carbamazepine and found comparable results, with a correlation coefficient of r ≈ 0.999, indicating that CMIA can reliably measure carbamazepine levels (Guerrero Garduño et al., 2016). Conversely, another study comparing HPLC and CMIA for valproic acid analysis indicated that HPLC offered greater precision than the immunoassay method (Zhao et al., 2016). Notably, highly metabolized drugs like cyclosporine A and tacrolimus were found to have significantly overestimated concentrations when measured by immunoassays compared to the UHPLC-MS method (Mei et al., 2018; Han et al., 2025).

Both CMIA and HPLC have been validated across various populations and for different drugs. Nonetheless, the potential for interference in CMIA necessitates careful interpretation of results, particularly in patients exhibiting altered pharmacokinetics.

Currently, there is a lack of data comparing the performance of CMIA with HPLC specifically for vancomycin, particularly in LTR patients. Therefore, this study aims to compare the accuracy and precision of CMIA and HPLC in measuring vancomycin levels in serum in LTRs, a topic that remains unexplored in the existing literature.

## 2 Methods

### 2.1 Study design, setting, and population

This cross-sectional study was done for 11 months, from January 2023 to November 2023, at the Abu-Ali Sina Solid Organ Transplant Hospital, affiliated with Shiraz University of Medical Sciences, Shiraz, Iran. The study was approved by the ethics committee of Shiraz University of Medical Sciences (IR.SUMS.REC.1400.021). All the study protocols were according to the ethical guidelines of the 1975 Helsinki Declaration. Informed consent was obtained from all study participants.

The studied population was patients having undergone liver transplantation and been under vancomycin treatment. Inclusion criteria required participants to be over 18 years old and to have received at least four doses of vancomycin. Excluded from the study were individuals with hypersensitivity to vancomycin, chronic kidney disease or on dialysis, burn injuries, those taking phenytoin, and pregnant women.

A combination of a calcineurin inhibitor (CNI) with sodium mycophenolate and prednisolone was prescribed for all patients as the immunosuppressive regimen. Dose adjustment was performed based on serum drug levels and the patient’s clinical response.

### 2.2 Data gathering

All necessary data was gathered through patients’ medical records. They included demographic and anthropometric characteristics (age, sex, height, Actual body weight (ABW), Ideal body weight (IBW), and Body mass index (BMI)), time since liver transplant, history of previous ICU admission, positive microbiologic cultures, type of infection, indication for transplantation, comorbidities, laboratory parameters (including, Blood urea nitrogen (BUN), Creatinine (Cr), Albumin (Alb), Total Bilirubin (T.Bili), Direct Bilirubin (D.Bili), Aspartate aminotransferase (AST), Alanine transaminase (ALT), Magnesium (mg), and concomitant antibiotics.

### 2.3 Vancomycin dosage and administration

The study protocol involved the administration of vancomycin to LTRs using a dosing regimen that included a loading dose of 20–35 mg/kg and a maintenance dose of 15–20 mg/kg, given every 12 h for at least four doses (Rybak et al., 2020). Infectious disease specialists were responsible for deciding whether to prescribe vancomycin empirically or definitively. The vancomycin infusion was given over a period of 60–90 min as per the study protocol, which aimed to evaluate the pharmacokinetics and outcomes associated with vancomycin use in LTRs.

### 2.4 Sampling

To ensure reaching the steady-state plasma concentrations, TDM should be started 48 h after initiation of vancomycin therapy. Four blood samples were taken from each participant at different time intervals, including 1) trough 1 (just before the fifth dose administration), 2) peak (5 min after the termination of the fifth dose infusion) 3) intermediate (6 h after the fifth dose administration), and 4) trough 2 (12 h after the fifth dose or just before the sixth dose administration).

### 2.5 Sample preparation and vancomycin CMIA analysis

At four mentioned times (0, 1, 6, and 12 h), a 4 mL sample was collected and decanted into K_2_EDTA tubes to prevent clotting. The plasma was separated from the whole blood by centrifuging the samples at 4,000 rpm for 3 min. The resulting plasma was then equally aliquoted for both CMIA and HPLC analysis.

According to the manufacturer’s instructions, vancomycin concentration in plasma was analyzed through the Vancomycin kit (The vancomycin assay on Architect i2000SR, Abbott^®^, North Chicago, 229 Illinois, United States). It was based on a reaction between plasma vancomycin and vancomycin kit reagents, reading the absorbance via an architect CMIA analyzer, and calculating the vancomycin concentration using the calibration curve provided with the Abbott^®^ kit. The detection range was 3.0–100.0.

### 2.6 Sample preparation and vancomycin HPLC analysis

A 50 µL solution of theophylline standard (with a concentration of 8 mg/L) was mixed with 950 µL of the plasma using a vortex at 2,000 rpm for 1 min. To extract vancomycin from the plasma and precipitate plasma proteins, 1,000 µL of methanol was added to the mixture. The mixture was then centrifuged at 12,000 rpm for 15 min, and the supernatant was subsequently analyzed using a validated HPLC method.

### 2.7 HPLC apparatus and conditions

The high-performance liquid chromatography (HPLC) system consists of a pump-controller unit and a UV detector was used for sample analysis (Knauer, Germany). Separation was fulfilled by isocratic elution with a mobile phase of phosphate buffer (pH of 2.2, 0.03 M) and acetonitrile (86:14 %v/v ratio), transferred at a flow rate of 0.72 mL/min through a C18 column (250 mm length × 4.6 mm I.D.; 5 μm pore size, Knauer, Germany) as the stationary phase. The chromatographic pattern was recorded at the wavelength of 205 nm and column temperature of 25°C.

### 2.8 Vancomycin-induced nephrotoxicity detection

According to the KDIGO (Kidney Disease: Improving Global Outcomes) guidelines, vancomycin-induced nephrotoxicity is diagnosed based on an increase in serum creatinine by ≥ 0.3 mg/dL within 48 h after vancomycin initiation or an increase in serum creatinine by ≥ 1.5 times baseline within 7 days after starting vancomycin therapy or a urine output <0.5 mL/kg/h for 6 h after vancomycin initiation (Roy et al., 2013).

Serum creatinine levels were measured before vancomycin administration, and daily monitoring of serum creatinine levels and creatinine clearance was conducted until hospital discharge to detect changes in kidney function and diagnose vancomycin-induced nephrotoxicity regarding the KDIGO criteria.

### 2.9 Pharmacokinetic variables

To determine pharmacokinetic parameters for each patient, four equations were used.

Equation 1 was applied to calculate systemic clearance of the vancomycin.

$$Cl=\frac{X_{0}}{\text{AUC}\tau }$$ (1)

Where Cl stands for clearance in L/h, X_0_ is the symbol of the administered dose in mg in each dosing interval, and AUCτ is the AUC of intervals in mg.h/L. AUCτ was computed through the trapezoidal method, which estimates the area by dividing it into trapezoidal sections, using four blood samples collected from each participant.

To align with recent guidelines (He et al., 2020), we calculated the AUC24 h for these patients by doubling the AUC12. This approach was adopted based on the absence of significant differences between the first and second trough concentrations in patients receiving vancomycin, indicating that drug accumulation was unlikely during the assessment period.

Equation 2 was applied to measure the volume of drug distribution within the body.

$$V_{d}=\frac{X_{0}}{{\text{C}}_{\text{max}}-{\text{C}}_{\text{min}}}$$ (2)

Where V_d_ stands for the volume of distribution in L, X_0_ is the symbol of the administered dose in mg in each dosing interval, and C_max_ and C_min_ show the peak and trough concentration in mg/L, respectively.

Equation 3 was applied to compute the elimination constant of vancomycin.

$$k=\frac{Cl}{V_{d}}$$ (3)

Where k is the symbol of the elimination constant in h−1, Cl stands for clearance in L/h, and V_d_ is the volume of distribution in L.

Equation 4 was applied to account for the elimination half-life of vancomycin.

$$t_{1/2}=\frac{0.693}{k}$$ (4)

Where t_1/2_ stands for vancomycin half-life in h and k is the elimination constant in h−1.

After each patient sample analysis, the doses were adjusted based on Equations 5, 6.

$$k_{0}=\frac{\left({\text{C}}_{\text{min }}\right)\left(V_{d}\right)\left(k\right)}{1-e^{-kƬ}}$$ (5)

Where k_0_ is the symbol of the administration rate in mg/h, C_min_ stands for the targeted trough concentration in mg/L, V_d_ shows the volume of distribution in L, k is the elimination constant in h−1, and τ is the dosing interval in h. (Assuming a one-compartment distribution model).

$$\frac{AUC2}{AUC1}=\frac{Dose2}{Dose1}$$ (6)

Where AUC_2_ is the target AUC, AUC_1_ is the current AUC, Dose2 is the new required dose to obtain the target AUC, and Dose1 is the current dose. (Assuming linear pharmacokinetic).

The seven following Equations 7–13 were applied to calculate trough and peak concentrations, the area under the curve, the volume of distribution, creatinine clearance, and the elimination rate constant.

$${\text{C}}_{\text{min}}={\text{C}}_{\text{max }}\left(e^{-kƬ}\right)$$ (7)

Where Cmin and Cmax are trough and peak concentrations at the steady state, respectively.

$${\text{C}}_{\text{max }}=\frac{\frac{Dose}{V_{d}}}{\left(1-e^{-kƬ}\right)}$$ (8)

$${\text{AUC}}_{Ƭ}=\frac{{AUC}_{24}}{\frac{24}{Ƭ}}$$ (9)

$${\text{AUC}}_{24}=\frac{Dose24h}{Clv}$$ (10)

Where Dose_24h_ stands for a total daily dose of vancomycin.

$$V_{d}=\left\{\begin{array}{c} {0.9\;L/\text{kg},\text{if}\;\text{Cl}}_{\text{cr}}\;<\;60\text{ ml}/\min\\ 0.72\;{L/\text{kg},\text{if}\;\text{Cl}}_{\text{cr}}\geq 60\text{ ml}/\min \end{array}\right.$$ (11)

Where Cl_cr_ stands for creatinine clearance in mL/min being a measure of kidney function and V_d_ is the volume of distribution in L which is dependent on Cl_cr_, itself.

$$\text{Clv}=\left(\text{Clcr}\times 0.693\right)+3.66$$ (12)

Where Cl_v_ stands for vancomycin clearance.

$$k=\frac{\ln\left(\frac{Cmax}{Cmin}\right)}{Ƭ}$$ (13)

Where k is the elimination rate constant in $h^{-1}$ .

### 2.10 Statistical analysis

All statistical analyses were accomplished using IBM Statistical Package for Social Sciences software, version 25 (IBM Corporation, NY, United States). Continuous variables were reported as mean ± SD or median ± IQR and categorical parameters were expressed as percentages. The normality of continuous variables was examined via the Kolmogorov-Smirnov test. To determine the association between concentration obtained from HPLC and CMIA analysis the univariate regression model was used. The assessment of the correlation between data of HPLC and CMIA was carried out through the Pearson correlation test, as well. A p-value of less than 0.05 was considered statistically significant for all the above analyses.

## 3 Results

### 3.1 Demographic characteristics

With consideration of eligible criteria, 34 adult liver transplant recipients were included. More than half of the patients (61.8%) were male. The mean ± SD age and weight of participants were 43.3 ± 2.05 years and 69.9 ± 2.32 kg, respectively. The leading causes of liver failure in these participants were autoimmune hepatitis, followed by viral hepatitis, primary sclerosing cholangitis (PSC), and non-alcoholic steatohepatitis (NASH), respectively. The median (median ± IQR) serum creatinine of patients before vancomycin initiation was 0.9 ± 0.52 mg/dL. Most (88.2%) patients received vancomycin during the first month after transplantation. The mean loading and maintenance dose of vancomycin were 1030.9 ± 25.15 mg and 714.7 ± 21.16 mg, respectively. The median time of vancomycin receiving was 5 ± 3 days. Fifteen patients (44.1%) experienced nephrotoxicity during the study period within vancomycin treatment. Table 1 summarizes the demographic characteristics of LTRs receiving vancomycin.

**TABLE 1:** Demographic characteristics in liver transplant recipients receiving vancomycin (N = 34).

Parameters	Total (N = 34)	Patients without vancomycin induced nephrotoxicity (N = 19)	Patients with vancomycin induced nephrotoxicity (N = 15)	P-value
Demographic data				
Age (year) (Mean ± SD)	43.26 ± 11.96	43.5 ± 12.7	42.9 ± 11.4	0.884
**Gender**				
Male, n (%)	21 (62)	13 (68.4)	8 (53.3)	0.371
Female, n (%)	13 (38)	6 (31.6)	7 (46.7)	
BMI (kg/m^2^) (Mean ± SD)	24.2 ± 3.8	23.6 ± 3.8	24.9 ± 3.9	0.336
Time since liver transplantation (days) (Mean ± SD)	5 ± 8	6 ± 14	5 ± 9	0.306
Previous ICU admission, n (%)	18 (53)	9 (47.4)	9 (60)	0.465
MELD Score (Mean ± SD)	24.5 ± 11.5	22.8 ± 8.8	26.8 ± 6.6	0.153
Indication for transplantation(N)				
NASH + Cryptogenic	7	4	3	0.940
PSC + PBC	7	3	4	0.440
HBV + HCV	3	2	1	0.696
Wilson	1	1	0	1.000
AIH	12	8	4	0.353
Others	4	1	3	0.210
Comorbidities(N)				
Diabetes Mellitus	4	3	1	0.426
Hypertension	0	0	0	-
CVD	0	0	0	-
COPD + Asthma	0	0	0	-
Hypothyroidism	2	2	0	0.999
Dyslipidemia	5	2	3	0.445
Laboratory parameters (mg/dL)				
Baseline BUN * (Median ± IQR)	12.5 ± 8.5	11 ± 8	13 ± 12	0.493
Baseline Cr * (Median ± IQR)	0.8 ± 0.3	0.8 ± 0.3	0.8 ± 0.3	0.503
BUN zero* (Median ± IQR)	21 ± 10.8	23 ± 15	19 ± 4	0.471
Cr zero* (Median ± IQR)	0.9 ± 0.52	0.9 ± 0.7	0.9 ± 0.4	0.221
BUN 7th day* (Median ± IQR)	13.5 ± 16.3	12 ± 10	25 ± 25	0.017
Cr 7th day* (Median ± IQR)	0.9 ± 0.3	0.9 ± 0.4	0.8 ± 0.6	0.551
Concomitant antibiotics(N)				
Aminoglycosides	5	2	3	0.445
Colistin	8	3	5	0.240
Carbapenems	10	5	5	0.656
Amphotericin B	3	2	1	0.696

Baseline: Before transplantation, Zero: before vancomycin initiation, seventh day: 7 days after vancomycin initiation.

Abbreviations: BMI, Body mass index; ICU, Intensive care unit; MELD, Model for end-stage liver disease; NASH, Nonalcoholic steatohepatitis; PSC, Primary biliary cirrhosis; PBC, Primary sclerosing cholangitis; HBV, Hepatitis B virus; HCV, Hepatitis C virus; AIH, Autoimmune hepatitis; CVD, Cardiovascular disease; COPD, Chronic obstructive pulmonary disease.

### 3.2 Pharmacokinetic outcome

According to Table 2, while the vancomycin concentrations measured through the HPLC method at intervals of 1 (trough1), 3 (intermediate), and 4 (trough2) were significantly higher in patients with vancomycin-induced nephrotoxicity, the vancomycin concentrations measured via CMIA method were statistically equal between the two groups at all intervals. In addition, among pharmacokinetic variables, T_1/2_, AUC_12h_, and AUC_24h_ observed based on the HPLC method exhibited significant differences between LTR patients with and without vancomycin-induced nephrotoxicity, where patients with reduced kidney function had significantly longer T_1/2_ and also higher AUC_12h_, and AUC_24h_. In spite of that, no significant association was detected between any pharmacokinetic variables observed through the CMIA method and developing vancomycin-induced nephrotoxicity. Furthermore, calculated pharmacokinetic parameters indicated no significant correlation with the emergence of nephrotoxicity. The analysis also exposed a non-significant association between the observed and calculated pharmacokinetic outcomes in LTRs, underscoring the indispensability of TDM for ascertaining kinetic parameters and dosage modifications.

**TABLE 2:** Comparison of pharmacokinetic outcome in liver transpalnt recipients that had been administered vancomycin through HPLC and CMIA method (N = 34).

Variables	Total (N = 34)	Non-vancomycin induced nephrotoxicity (N = 19)	Vancomycin induced nephrotoxicity (N = 15)	P value
Concentration (mg/L)				
HPLC concentrations				
C1 (Trough1)	11.3 ± 6.3	9.1 ± 5.3	14.1 ± 6.4	0.026
C2 (Peak)	24.4 ± 12.2	21.3 ± 9.3	28.4 ± 14.4	0.101
C3 (Intermediate)	14.3 ± 8.1	11.8 ± 7.9	17.5 ± 7.3	0.049
C4 (Trough2)	11.8 ± 6.1	9.3 ± 4.9	15.0 ± 6.1	0.011
CMIA concentrations				
C1 (Trough1)	16.46 ± 14.21	13.12 ± 13.83	20.67 ± 14.51	0.157
C2 (Peak)	46.96 ± 22.86	45.81 ± 20.56	48.42 ± 26.85	0.741
C3 (Intermediate)	21.00 ± 10.62	18.79 ± 8.00	23.94 ± 13.24	0.174
C4 (Trough2)	18.40 ± 15.12	17.41 ± 13.24	19.64 ± 12.48	0.671
Pharmacokinetic variables				
Observed (based on HPLC method)				
K (1/h)	0.08 ± 0.04	0.08 ± 0.03	0.07 ± 0.06	0.287
$T_{\frac{1}{2}}$ (h)	12.2 ± 6.6	9.7 ± 4.2	15.3 ± 7.9	0.024
Vd (L)	76.26 ± 54.25	70.0 ± 28.4	84.2 ± 77.5	0.467
Vancomycin clearance (L/h)	4.9 ± 3.0	5.5 ± 2.4	4.2 ± 3.6	0.206
AUC12 h (mg.h/L)	191.65 ± 96.15	159.84 ± 83.96	231.95 ± 98.06	0.037
AUC24 h (mg.h/L)	383.3 ± 192.3	319.7 ± 167.9	463.9 ± 196.1	0.037
Observed (based on CMIA method)				
K (1/h)	0.11 ± 0.06	0.120 ± 0.065	0.081 ± 0.064	0.109
$T_{\frac{1}{2}}$ (h)	9.53 ± 18.15	6.29 ± 2.74	13.43 ± 26.52	0.761
Vd (L)	37.33 ± 75.60	26.46 ± 17.83	50.37 ± 111.1	0.734
Vancomycin clearance (L/h)	2.93 ± 2.02	2.84 ± 1.23	3.04 ± 2.81	0.781
AUC12 h (mg.h/L)	317.81 ± 150.80	297.02 ± 122.53	344.80 ± 185.62	0.364
AUC24 h (mg.h/L)	635.61 ± 301.60	594.04 ± 245.06	689.60 ± 371.2 4	0.364
Calculated				
K (1/h)	0.087 ± 0.03	0.084 ± 0.03	0.092 ± 0.03	0.499
$T_{\frac{1}{2}}$ (h)	9.55 ± 5.07	10.55 ± 6.40	2.02 ± 2.61	0.233
Vd (L)	51.64 ± 9.7	51.91 ± 9.74	51.31 ± 10.42	0.858
Vancomycin clearance (L/h)	4.4 ± 1.6	4.56 ± 1.50	4.14 ± 1.72	0.31
AUC12 h (mg.h/L)	187.46 ± 78.75	205.41 ± 95.47	164.72 ± 48.48	0.43
AUC24 h (mg.h/L)	374.92 ± 157.50	410.82 ± 190.9	329.44 ± 96.96	0.154

HPLC, High-performance liquid chromatography; CMIA, Chemiluminescent microparticle immunoassay; T_1/2_, Half-life; V_d_, Volume of distribution; AUC, Area under the curve; K, elimination rate constant.

### 3.3 The correlation and association between HPLC and CMIA concentrations

Regarding low R square values shown in Table 3, there was no linear regression between concentrations obtained by HPLC and CMIA. Also, no significant correlation was detected between these two variables.

**TABLE 3:** The association between HPLC and CMIA concentrations among liver transpalnt recipients using linear regression. (N = 34).

C1 HPLC and C1 CMIA	R Square	0.33
	Adjusted R square	0.31
	Confidence interval	0.15–0.47
	Pearson correlation	0.62
C2 HPLC and C2 CMIA	R Square	0.13
	Adjusted R square	0.11
	Confidence interval	0.02–0.42
	Pearson correlation	0.35
C3 HPLC and C3 CMIA	R Square	0.36
	Adjusted R square	0.34
	Confidence interval	0.22–0.64
	Pearson correlation	0.58
C4 HPLC and C4 CMIA	R Square	0.34
	Adjusted R square	0.32
	Confidence interval	0.13–0.39
	Pearson correlation	0.45

HPLC, High-performance liquid chromatography, CMIA, Chemiluminescent microparticle immunoassay; C1, Trough1; C2, Peak; C3, Intermediate; C4, Trough2.

### 3.4 Comparison of CMIA and HPLC concentrations in the studied population

**FIGURE 1:**
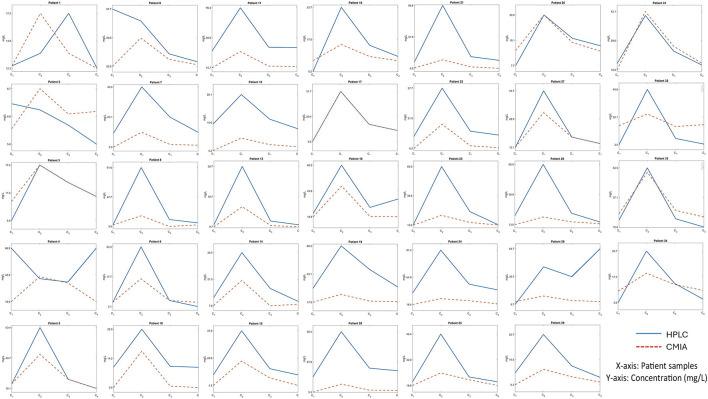
The vancomycin concentration curve in a 12-hour-period of blood sampling for 34 liver transplant recipients using both the CMIA and HPLC methods.

**FIGURE 2:**
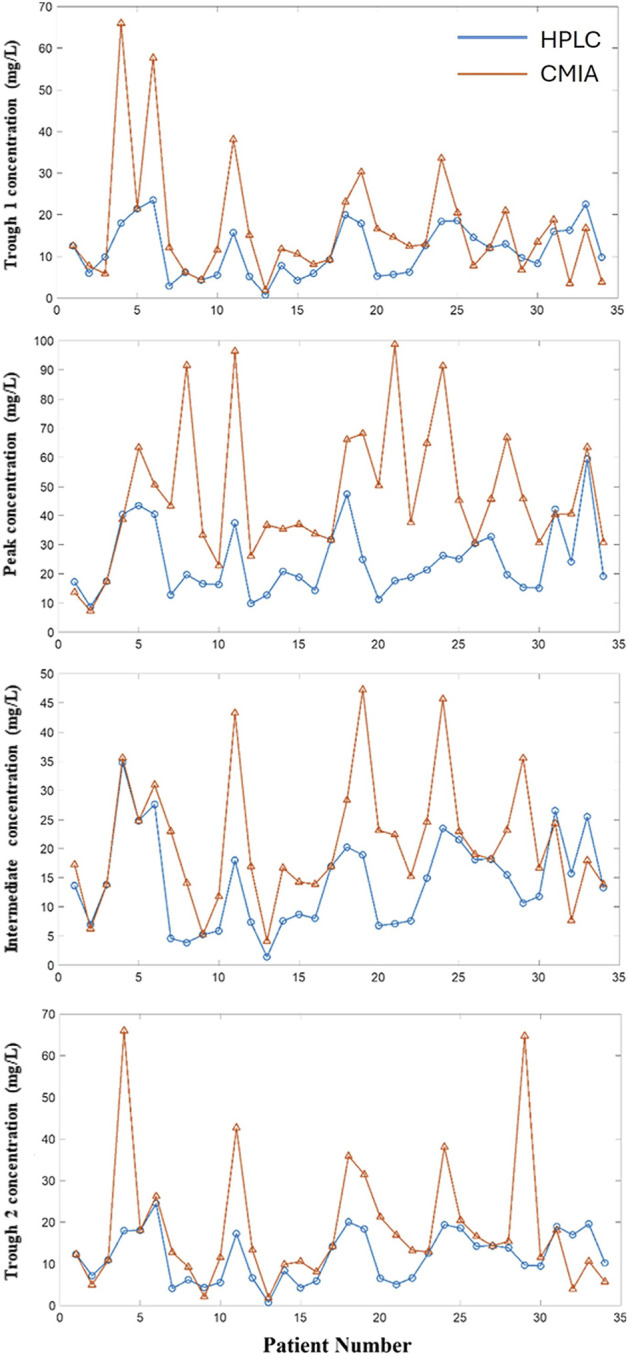
Comparison of different concentrations via HPLC and CMIA methods among 34 liver transplant recipients.

The study investigated the vancomycin concentration profile over 12 h through blood sampling from 34 patients, using both the CMIA and HPLC methods (Figure 1). The results revealed significant differences between the two methods, particularly in peak concentration levels (Figure 2). As detailed in Table 4, the mean peak concentrations obtained via the CMIA and HPLC methods were 46.96 ± 23.20 mg/L and 22.94 ± 14.09 mg/L, respectively, with a p-value of <0.0001. Additionally, the trough concentrations also exhibited significant differences between the two methodologies (p-values: 0.02 and <0.000). The CMIA method reported higher first and second trough concentrations (17.55 ± 14.73 mg/L and 17.26 ± 13.40 mg/L, respectively) compared to the HPLC method (11.79 ± 7.7 mg/L and 9.03 ± 5.79 mg/L, respectively). Therefore, the study concluded that the CMIA method had low accuracy in measuring vancomycin concentrations and therefore recommended the use of TDM to ascertain kinetic parameters and dosage modifications.

**TABLE 4:** The average concentrations and differences between HPLC and CMIA among liver transplant recipients (N = 34).

		C1	C2	C3	C4
Mean ± SD (mg/L)	CMIA	17.55 ± 14.73	46.96 ± 23.20	21.09 ± 10.93	17.26 ± 13.40
	HPLC	11.79 ± 7.7	22.94 ± 14.09	11.75 ± 8.1	9.03 ± 5.79
Mean differences (%)		49	105	79	91
p-value		0.02	<0.0001	<0.0001	<0.0001

HPLC, High-performance liquid chromatography; CMIA, Chemiluminescent microparticle immunoassay; C1, Trough1; C2, Peak; C3, Intermediate; C4, Trough2.

## 4 Discussion

Therapeutic vancomycin monitoring is essential for the optimization of its therapeutic effect and the avoidance of nephrotoxicity. Several methods have been developed for this purpose, including HPLC and CMIA. CMIA is a rapid, simple, automated, and inexpensive method that has been highly taken into consideration. However, the main concern of using this method can be the accuracy and precision of the measurement. This is the first study comparing HPLC and CMIA methods to assess vancomycin concentration in LTRs.

Currently, the existing guidelines do not provide specific dosing regimens for particular populations, such as LTRs. Therefore, it is essential to first calculate the Pharmacokinetic parameters within this population before determining the most optimal dosage.

None of the pharmacokinetic variables observed from the CMIA and HPLC concentrations have a significant relationship with the calculated variables in LTR and it emphasizes the need to use therapeutic monitoring.

The present study used an accurate, selective, and sensitive validated HPLC method (Ghasemiyeh et al., 2020) and Abbott^®^ kit for vancomycin quantification. The limit of detection for HPLC and CMIA methods were 800 ng/mL and 3 μg/mL, respectively. So, HPLC provides a more sensitive assay for vancomycin.

Our results showed that vancomycin concentration obtained by HPLC and CMIA presented no linear regression between the two methods and the HPLC method was considered more accurate, precise, and versatile for both pharmacokinetic analysis and distinguishing the concentration differences between the patients with and without vancomycin-induced nephrotoxicity, significantly in trough 1, intermediate, and trough 2 concentrations. Considering the comparison of CMIA and HPLC concentrations in the studied population, the concentration investigated by CMIA was higher than that by HPLC in almost all patients during the 12-hour sampling. The peak concentration indicated the greatest differences and had a 105% deviation on average. Fan et al. have also reported an overestimated concentration via CMIA compared to UPLC/MS among hemodialysis patients, possibly secondary to interference of various panels of serum samples (Fan et al., 2019). Furthermore, a case report study serendipitously discovered a falsely high level of vancomycin using immunoassay methods in a patient not actually receiving this medication, perhaps due to being affected by endogenous proteins cross-interaction (Tsoi et al., 2019). However, Scribel et al. reported no cross-reactivity via CMIA among patients on hemodialysis (Scribel et al., 2024). On the other hand, numerous studies have examined the link between vancomycin trough levels and the risk of nephrotoxicity, generally finding that higher trough levels correlate with increased nephrotoxicity rates. In this regard, Horey et al. observed a correlation between vancomycin trough levels and nephrotoxicity rates. They found that the rates of nephrotoxicity were 4.9%, 3.1%, 10.6%, 23.6%, and 81.8% for maximal troughs of 5–10 mg/L, 10.1–15 mg/L, 15.1–20 mg/L, 20.1–35 mg/L, and greater than 35 mg/L, respectively (Horey et al., 2012). Similarly, Lodise et al. revealed that patients with trough levels greater than 10 mg/mL significantly more experienced nephrotoxicity compared to trough levels less than 10 mg/mL (22% vs. 5.1%) (Lodise et al., 2009). Cano et al. also noted an increase in nephrotoxicity from 7% at levels below 10 mg/L to 34% at levels above 20 mg/L (Cano et al., 2012). Barriere et al. observed no renal adverse events in patients with trough levels below 10 mg/L, while those with levels above this threshold experienced increasing incidences of renal issues (3% for 10–15 mg/L and 17% for above 15 mg/L) (Barriere et al., 2014). The present study similarly indicated that LTR patients having developed vancomycin-induced nephrotoxicity had a significantly higher vancomycin concentration at trough 1 (C1), intermediate (C3), and trough 2 (C4) observed via HPLC, however, the CMIA method was not accurate enough to distinguish the significant differences between the two groups at any intervals. In addition, the data obtained by HPLC showed that trough concentrations higher than 10 mg/L were associated with an increased risk of acute kidney injury in LTRs, highlighting the importance of monitoring pharmacokinetic parameters in this population to reduce adverse effects.

LTRs may experience altered pharmacokinetic parameters compared to the normal population due to the post-transplant inflammation state, changes in plasma protein level, the presence of hepatorenal syndrome, postoperative complications, and polypharmacy (Morales Junior et al., 2023; Righi, 2018; De Simone et al., 2024). The mean V_d_ in our investigation measured by HPLC and CMIA were 1.1 ± 0.7 and 0.53 ± 1.08 L/kg, respectively. V_d_ obtained from CMIA was in the typical range of 0.4 L/kg to 1 L/kg previously observed in the normal populations, while the value obtained from HPLC was significantly higher than this range which was also noted by other studies (Zhang et al., 2020) Among the studied population, many inter-individual variations were observed in V_d_ values. In this regard, the lack of significant correlation between the calculated and observed V_d_ highlights the possible changes due to fluid retention caused by organ dysfunction, enhanced capillary permeability resulting from systemic inflammatory response syndrome (SIRS) in ICU admitted patients, and ascites presence as a third-space volume. Additionally, lower baseline albumin levels in liver transplant recipients can also contribute to an increase in volume of distribution (Vd), particularly for hydrophilic antibiotics such as vancomycin. This effect was also emphasized in ICU-admitted critically ill patients in the study of Polard et al. (1999), in pediatric LTRs in the study of Shoji et al. (2021), and in adults having undergone bone marrow transplantation in the study of Taghizadeh Ghehi et al. (2013). Our findings demonstrated an increase in vancomycin clearance in LTRs, with a mean clearance of 4.91 ± 3.00 L/h and 2.93 ± 2.02 L/h obtained from HPLC and CMIA, respectively, compared to the general population’s average of 2.6 L/h. This elevation in clearance can be attributed to lower levels of albumin in LTRs, leading to higher unbound vancomycin and increased total clearance (both renal and non-renal) (Flannery et al., 2020; Rodvold et al., 1988), which are more manifest in data measured via the HPLC method in our study. Furthermore, the inflammatory condition prevalent in post-transplant recipients and also the use of diuretic drugs in such patients can exacerbate the renal excretion of antibiotics, leading to enhanced vancomycin clearance (Shimamoto et al., 2013). Moreover, we obtained a longer t_1/2_ for vancomycin in LTRs than normal population. Harada H et al. have also found that patients with hepatic impairment exhibit a prolonged vancomycin t_1/2_ (Harada et al., 1999). Additionally, there was a significant increase in t_1/2_ in LTRs with renal insufficiency compared to non-vancomycin-induced nephrotoxicity LTRs through HPLC measurement, while CMIA data demonstrated no differences. Cheung et al. study reported longer vancomycin half-life in patients with impaired renal function, as well (Cheung and DiPiro, 1986). Our study also revealed a lower k_1/2_ in LTRs than the normal population due to changes in both Cl_v_ and V_d_ values. In addition, our AUC_24h_ and AUC_12h_ values were below therapeutic levels reported in previous studies, denoting that higher loading doses may be necessary to achieve therapeutic AUC in LTRs. According to recent guidelines from ASHP, IDSA, and SIDP, the recommended AUC for effective treatment of MRSA infections is generally considered to be ≥400 mg·hr/L, with some studies suggesting that higher AUC values may be necessary for more severe infections, Nevertheless, there have been no reports on the optimal dosing required to ensure adequate drug exposure in LTRs (Rybak et al., 2020; Shoji et al., 2021). In a study by Alvarez et al. on critically ill patients admitted to ICU has been demonstrated that patients receiving a loading dose exhibited an AUC ranging between 600 and 700 mg.h/L (Álvarez et al., 2017). Holmes et al. conducted another study on patients with S.aureus bacteremia, indicating that 54% of patients with hepatic impairment or receiving immunosuppressive agents had an AUC greater than 400 mg.h/L (Holmes et al., 2013). Brown et al. also performed a study on patients with infective endocarditis and complex MRSA bacteremia, which demonstrated that the mortality rate was significantly lower in patients whose AUC/MIC values exceeded 400 mg.h/L (Brown et al., 2012). In our present study, we found that 41.2% of LTRs had an AUC above 400 mg.h/L, and failure to achieve the target AUC was associated with higher mortality rates. The average AUC was 383.3 ± 192.3 and 635.61 ± 301.60 via HPLC and CMIA measurement, respectively. When HPLC was used to assay AUC, LTR patients with higher values were significantly more susceptible to developing vancomycin-induced nephrotoxicity. Accordingly, the two points should be considered. First, HPLC is a more reliable test for vancomycin assessment than CMIA. Second, higher loading doses may be necessary for the LTRs to achieve therapeutic AUC levels. The pharmacokinetic alteration after liver transplantation indicates that standard dosing regimens may not achieve adequate therapeutic levels of the drug, necessitating tailored dosing protocols to ensure optimal drug exposure. Such adjustments are essential for the effective management of severe MRSA infections while also minimizing the risk of toxicity (Shoji et al., 2021). Prolonged exposure to suboptimal vancomycin levels can lead to the development of microbial resistance particularly in MRSA infections (Nyandoro et al., 2025), while higher vancomycin exposure particularly when AUC exceeds 550 mg.h/L is associated with nephrotoxicity in the treatment of MRSA bacteremia (Lodise et al., 2020; Chiu and Sarwal, 2023). Notably, our study highlighted that patients not reaching the AUC target had a higher mortality rate, emphasizing the need for optimized dosing strategies in this population. Clinicians should adopt an individualized approach to vancomycin dosing in LTRs through routine TDM, considering factors like renal function, concurrent medications, and nutritional status. Also, the potential overestimation of concentration using the CMIA method may lead to errors in drug dose adjustments and negatively impact therapeutic outcomes if this method is employed.

Furthermore, this study presents several limitations that must be acknowledged when interpreting the findings regarding therapeutic vancomycin monitoring in LTRs. Firstly, the sample size of our study was relatively small, which may limit the generalizability of the results. A larger sample size would provide more robust data and potentially reveal additional insights into the pharmacokinetics of vancomycin in this specific population. Secondly, this research was conducted at a single center, which may introduce biases related to local practices, patient demographics, and clinical protocols. The findings may not be applicable to other institutions with different patient populations or treatment approaches. Multi-center studies would be beneficial to validate our results across diverse settings. Additionally, the focus of this study was exclusively on liver transplant recipients, which restricts the applicability of the findings to other transplant populations, such as kidney or heart transplant patients. Variations in drug metabolism and pharmacodynamics among different transplant types could yield different therapeutic monitoring requirements. Lastly, while HPLC is a reliable method, it lacks the sensitivity of techniques such as LC-MS, which could provide more precise vancomycin measurements, particularly in patients with fluctuating drug concentrations.

## 5 Conclusion

In conclusion, this study highlighted the importance of selecting the appropriate analytical method for TDM of vancomycin in liver transplant recipients. Our findings suggested that HPLC was a more sensitive and reliable method compared to CMIA for both measuring vancomycin concentration and detecting variable changes in its pharmacokinetic profile. The use of HPLC may help clinicians make more informed decisions regarding dosing adjustments and ultimately improve patient outcomes. But, while this study provides valuable insights into therapeutic vancomycin monitoring, the aforementioned limitations should be considered when interpreting the results and their implications for clinical practice. Further research addressing these limitations is warranted to enhance our understanding and explore other potential benefits of using HPLC for vancomycin TDM in LTRs.

## Data Availability

The raw data supporting the conclusions of this article will be made available by the authors, without undue reservation.
